# A Rapid Label-Free Fluorescent Aptasensor PicoGreen-Based Strategy for Aflatoxin B_1_ Detection in Traditional Chinese Medicines

**DOI:** 10.3390/toxins10030101

**Published:** 2018-02-28

**Authors:** Cheng Zhang, Xiaowen Dou, Lei Zhang, Meifeng Sun, Ming Zhao, Zhen OuYang, Dandan Kong, F. Logrieco Antonio, Meihua Yang

**Affiliations:** 1 Key Laboratory of Bioactive Substances and Resources Utilization of Chinese Herbal Medicine, Ministry of Education, Institute of Medicinal Plant Development, Chinese Academy of Medical Sciences, Peking Union Medical College, Beijing 100193, China; zcmycotoxin@163.com (C.Z.); douxiaowen573@163.com (X.D.); zhanglei-85@163.com (L.Z.); Marksmf@163.com (M.S.); k_dandan@163.com (D.K.); 2 School of Pharmacy JiangSu University, Zhenjiang 212013, China; yxzm@ujs.edu.cn (M.Z.); zhenouyang@ujs.edu.cn (Z.O.Y.); 3 National Research Council of Italy, CNR-ISPA, Via G. Amendola, 122/O, I-70126 Bari, Italy; antonio.logrieco@ispa.cnr.it

**Keywords:** aflatoxin B_1_, aptamer, PicoGreen, fluorescence, traditional Chinese medicines

## Abstract

Aflatoxin B_1_ (AFB_1_) is a very hazardous carcinogen, readily contaminating foodstuffs and traditional Chinese medicines (TCMs) that has inspired increasing health concerns due to dietary exposure. Colloidal nanocrystals have been proposed as optical labels for aptasensor assembly, but these typically require tedious multistep conjugation and suffer from unsatisfactory robustness when used for complex matrices. In the present study, we report a rapid and sensitive method for screening for trace AFB_1_ levels in TCMs using a label-free fluorescent aptasensor PicoGreen dye-based strategy. Using PicoGreen to selectively measure complementary double-stranded DNA, fluorescence enhancement due to dsDNA is ‘turned off’ in the presence of AFB_1_ due binding of aptamer target over complementary sequence. Self-assembly of a label-free fluorescent aptasensor based on AFB_1_ aptamer and PicoGreen dye was performed. Due to competition between the complementary sequence and AFB_1_ target, this rapid method was capable of highly sensitive and selective screening for AFB_1_ in five types of TCMs. This proposed approach had a limit of detection as low as 0.1 μg·L^−1^ and good linearity with a range of 0.1–10 μg·L^−1^ (0.1–10 ppb). Among the 20 samples tested, 6 batches were found to be contaminated with AFB_1_ using this method, which was confirmed using sophisticated liquid chromatography-electrospray ionization-tandem mass spectrometry/mass spectrometry analysis. The results of this study indicate the developed method has the potential to be a simple, quick, and sensitive tool for detecting AFB_1_ in TCMs.

## 1. Introduction

Aflatoxins, primarily produced by *Aspergillus flavus* and *A. parasiticus*, were defined as Group 1 human carcinogens by the International Agency for Research on Cancer in 1993 [1]. Highly lipophilic mycotoxins, aflatoxins can be quickly absorbed in the alimentary canal. It is estimated that 55 million of people worldwide have been exposed in an uncontrolled manner to aflatoxins through their diet [2,3]. In addition, aflatoxins can enter the bloodstream directly through inhalation [4]. There has been an increasing incidence of hepatomas associated with aflatoxin B_1_ (AFB_1_) that has been inspiring significant concern. AFB_1_ has been shown to have a variety of biological activities, including causing acute toxicity, stunting growth, being teratogenic, mutagenic, immunosuppressive, and genotoxic, and damaging membranes by increasing lipid peroxidation and generation of free radicals [5,6,7]. In view of the serious effects on humans of and pollution risk by this mycotoxin, governments and related organizations have established strict regulations and detection methods to limit aflatoxin levels in foodstuffs.

Currently, there is a range of methods available for detecting aflatoxins. These methods typically involve thin layer chromatography [8], high performance liquid chromatography (HPLC) coupled to fluorescent detectors [9], liquid chromatography coupled to mass spectrometry (MS) [10,11], and enzyme-linked immunosorbent assays (ELISA) [12]. However, the thin layer chromatography method is cumbersome, has low sensitivity, and requires a large amount of organic reagent. High performance liquid chromatography coupled to fluorescent detectors and liquid chromatography coupled to mass spectrometry rely on pre- or post-derivatization and a sophisticated mass spectrometer prior for detection, respectively. In addition, these methods require tedious pretreatments, making screening more difficult and increasing analysis time. Despite the rapidity and simplicity of classical enzyme-linked immunosorbent assays, these can have false positive results, large deviations in qualitative results, and matrix interference [13,14,15]. Antibody-based bioanalysis to detect contaminants requires laborious, expensive, and time-consuming antibody preparation and may be susceptible to problems with stability or modification. Therefore, rapid, simple, sensitive, and cost-effective methods are highly desired for quantifying AFB_1_ levels in matrices, including traditional Chinese medicines (TCMs).

To overcome the above limitations, aptamers (Apt) have been developed as novel recognition molecules promising for use in analytical applications [16]. Aptamers are single-stranded DNA or RNA molecules that can bind with high affinity and specificity to various target ligands, including small-molecule drugs, peptides, proteins, and cells. These molecules are selected in vitro using systematic evolution of ligands by exponential enrichment [17,18]. In addition to their strong target-binding ability, aptamers offer several notable advantages over traditional protein antibodies, including target diversity, high stability, and easy synthesis and modification for applications [19,20]. A variety of aptamer-based analytical techniques have been developed, including colorimetric assays, fluorescence assays, and electrochemical aptasensor methods [21]. Fluorescence-based analysis is particularly appealing due to its high sensitivity and the variety of flexible platforms available [22,23,24,25,26,27]. These fluorescence platforms can be categorized into those involving labeled [27] and label-free aptasensors [24]. Labeled aptasensors have good stability, low limits of detection, and wide detection ranges. However, these molecules are also complicated to couple, difficult to purify, and not conducive to rapid detection. By contrast, label-free aptasensor strategies have garnered much attention due to not requiring complex coupling steps, having stable signals and low matrix interference, and the capacity for use in rapid detection.

In this present study, we developed a facile and label-free aptasensor strategy for highly sensitive and selective fluorescence detection of AFB_1_ using commercially available PicoGreen (PG). Briefly, the fluorescent signal of PG generated upon binding to double-stranded DNA (dsDNA) formed from an aptamer and its complementary sequence (Seq) was turned off in the presence of AFB_1_ due to AFB_1_ recognition by the aptamer prior to the Seq. This assay had high sensitivity and has great potential for use for on-site and rapid analysis, which is conducive to detecting mycotoxins in TCMs.

## 2. Results and Discussion

### 2.1. Principle Underlying Label-Free Aptasensor Strategy

**Figure 1:**
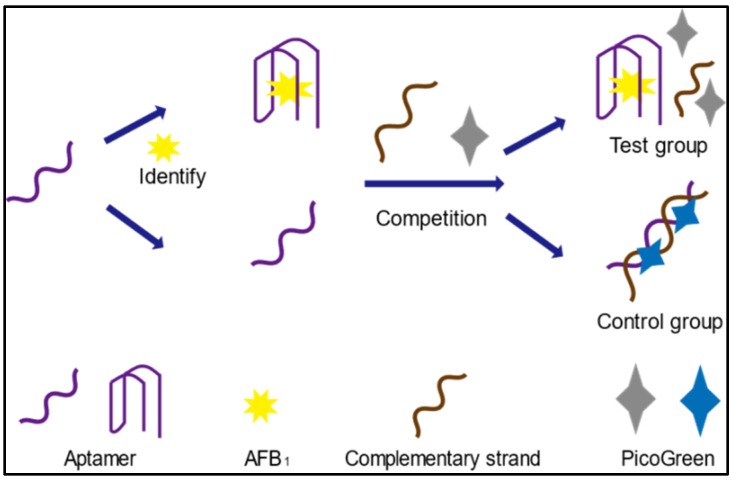
Schematic illustration of fluorescent detection of AFB_1_ by a label-free aptasensor.

**Figure 2:**
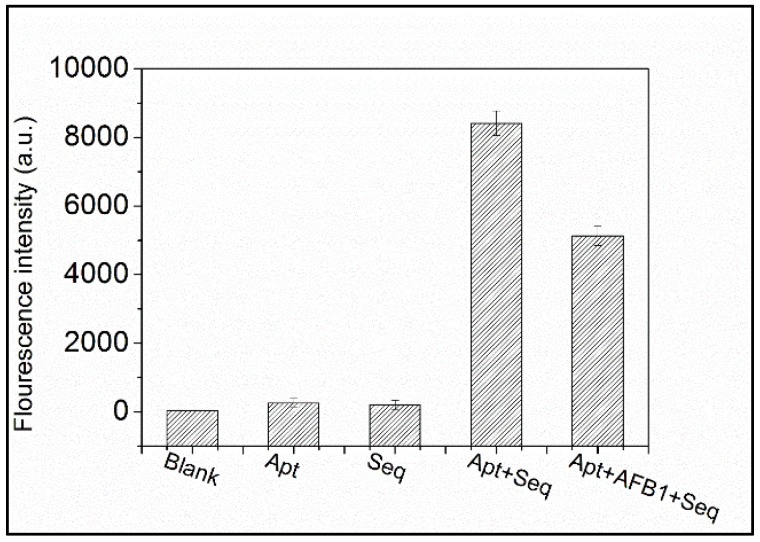
Principle of the label-free fluorescent aptasensor strategy of AFB_1_ detection. Results from three independent experiments.

PG reagent is an asymmetric cyanine dye that alone does not fluoresce. However, after binding to dsDNA, the fluorescence of this dye increases by more than 1000-fold [28]. A sensitive and quantitative probe, this commercially available dye allows for the detection of dsDNA levels as low as 25 ng L^−1^. Utilizing PG’s excellent selectivity and sensitivity for trace dsDNA, a rapid and straightforward label-free aptasensor strategy based on competitive recognition of aptamer by AFB_1_ and single-stranded DNA (ssDNA) was proposed for small target screening. The sensing mechanism of the proposed method is illustrated in Figure 1. In the control group, Seq hybridized with free AFB_1_-specific aptamer. After the formation of dsDNA, PG inserts into the minor groove of the dsDNA duplex, resulting in significant enhancement of fluorescence [29]. In the test group, AFB_1_ bound to aptamer prior to dsDNA formation, leaving only a small about of aptamer remaining for Seq to react with, thus causing a decrease in PG fluorescence intensity compared to the control group. Based on this decrease in fluorescence intensity, AFB_1_ could be quantified. Importantly, a critical precondition of this methodology is the fluorescent intensity could not be increased by the targets, Seq, or aptamer. This was examined and the results are presented in Figure 2. The blank solution produced negligible fluorescence, while the aptamer and Seq each exhibited very weak fluorescence, likely from the presence of dsDNA duplexes within regions of the ssDNA. As shown in Figure 2, when both aptamer and Seq were present in solution, there was a strong fluorescent signal. Upon addition of the target, this fluorescence dramatically decreased in intensity. This phenomenon was perfectly in line with our hypothesis. In the presence of AFB_1_, AFB_1_ bound to the aptamer in advance of the complementary sequence, greatly reducing the chance of dsDNA formation and leading to a decrease in fluorescence. To further validate the proposed method, a mismatch experiment was conducted, and the results are presented in Figure S1. In this experiment, Apt1 recognized AFB_1_ and was used in the mismatch test. Various concentrations of AFB_1_ were mixed with Apt1, then identical amounts of Seq1, 2, and 6 (a total of 80 bases) were individually added to the above mixture and the intensity of each well was recorded. Apt1 and Seq1 sequences were completely complementary, while Seq2 and 6 were only partially complementary to Apt1. Fluorescence in the Apt1+Seq1 group decreased gradually as AFB_1_ content increased, while Apt1+Seq2 and Apt1+Seq6 caused irregular and negligible changes in fluorescence intensity. These results support that the proposed label-free aptasensor strategy is promising for quantitative analysis and effective and suitable for AFB_1_ detection.

### 2.2. Screening of AFB_1_-Specific Aptamers Using Aptasensor Strategy

**Figure 3:**
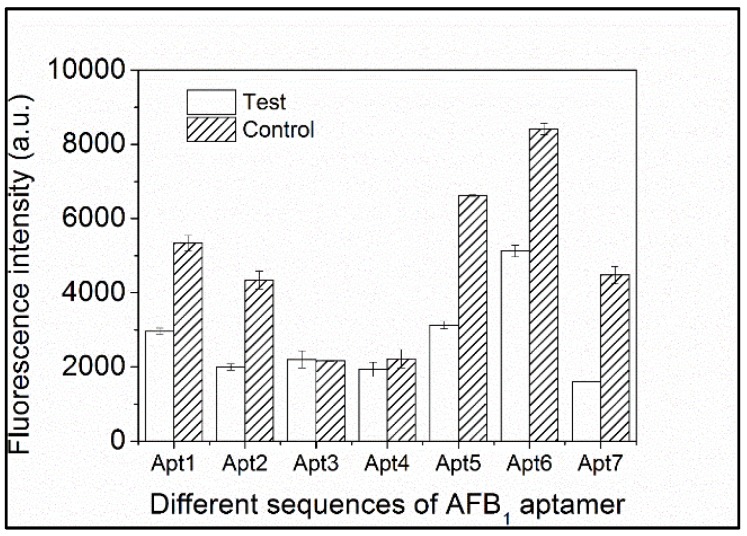
Evaluation of selectivity of aptasensor for AFB_1_ (using aptamers with different sequences).

Affinity and selectivity are bioanalysis foundations. In recent years, a number of aptamers have been selected for AFB_1_ recognition. Therefore, a total of seven aptamers (list in the Table 1) against AFB_1_ previously reported in the literature, as well as commonly used and patented sequences relating to selection or affinity studies, were investigated in this study. Aptamers have been described with defined dissociation constants (Kds). While Kd can be used to identify strong binding between aptamers and a small target, it fails to accurately describe specificity and cross-reactivity. Therefore, we used a simple and rapid method to screen for suitable aptamers using a commercial fluorescent dye. The results are shown in Figure 3. Compared with the corresponding control groups, the test groups containing AFB_1_ and Apt1, 2, 5, 6, and 7 had significantly less fluorescence intensity, indicating these aptamers strongly and selectively bound to AFB_1_ under these conditions. In particular, Apt5 bound the strongest with a distinct fluorescence intensity difference value (ΔF) of 3488.85 a.u. While Apt3 and 4 had good selectivity toward AFB_1_, neither displayed a decrease in fluorescence during the experiment in this study. Based on the notable difference in fluorescence intensity, Apt5 was used with non-labeled fluorescent dye PG to detect dsDNA. 

### 2.3. Specificity of the Label-Free Fluorescent Aptasensor for AFB_1_

**Figure 4:**
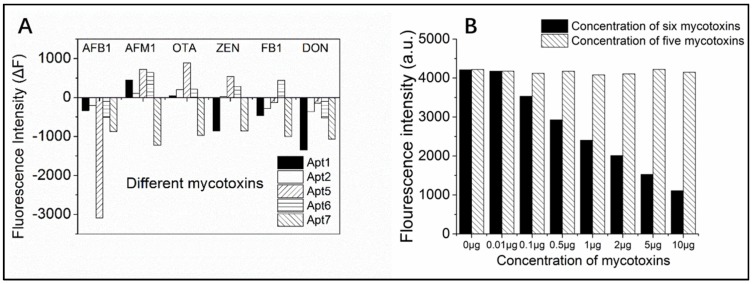
Label-free aptasensor using PG for detection of AFB_1_. (**A**) Evaluation of aptasensor specificity for AFB_1_ compared to AFM_1_, OTA, ZEN, FB_1_, and DON; (**B**) evaluation of aptasensor specificity for AFB_1_ in mixed standard solution (the black bar contained AFB_1_, another bar without AFB_1_).

The specificity of this assay for AFB_1_ and its analogues, aflatoxin M_1_ (AFM_1_), ochratoxin A (OTA), zearalenone (ZEN), fumonisin B_1_ (FB_1_), and deoxynivalenol (DON), was assessed using the proposed aptasensor strategy. As shown in Figure 4A, cross-reactivity was evaluated with 5 μg·L^−1^ of the aforementioned mycotoxins. AFB_1_ had the highest ΔF, while the other five analogues had a low ΔF, when detected with Apt5. This indicated Apt5 bound to AFB_1_ the strongest and had only slight binding with other mycotoxins. In particular, AFM_1_ contains a carbon skeleton identical to that in AFB_1_ except for a hydroxyl group on a C atom. However, the ΔF of AFB_1_ was still up to five-fold higher than that of AFM_1_ when using Apt5 as the recognition sequence. Based on these results, the other four aptamers tested (Apt1, 2, 6, and 7) were also able to recognize AFB_1_, but had much poorer specificity than Apt5. For example, the ΔF for Apt7 obtained for the six tested mycotoxins were almost identical. Moreover, as shown in Figure 4B, when AFB_1_ existed in the mixed standard solution, the fluorescence intensity decreased with increasing in concentration of AFB_1_. On the contrary, the variation was not obvious. For trace detection methods, significant cross-reaction can limit practical applications. In order for our assay to be sensitive enough to detect AFB_1_ in TCMs, Apt5 was used in follow-up experiments.

### 2.4. Optimization of Detection Conditions

#### 2.4.1. PG Amount

The assay established in this study was based on a combination of fluorescent dye and dsDNA. Therefore, the amount of fluorescent dye used is an important parameter in detection performance. Different PG solution amounts (5, 10, 15, 20, and 25 μL) were introduced to the mixture of Apt and Seq. As shown in Figure S2, the fluorescence intensity was directly correlated with PG, peaking when 15 μL PG was added. Under a constant quantity of Apt and Seq, increasing the PG did not lead to an intensity increase, supporting that 15 μL PG was enough to mark dsDNA after binding of complementary strands. 

#### 2.4.2. PG and dsDNA Reaction Time

Our approach relies on changes in PG fluorescence intensity in response to competitive binding to Apt between Seq and AFB_1_. AFB_1_ had priority for binding to Apt, while the subsequent Seq probably competitively combined with Apt from the Apt-AFB_1_ adduct during the reaction time. Therefore, it is important to determine the optimal time for dsDNA formation and PG labeling. As shown in Figure S3, the intensities for all AFB_1_ concentrations first increased and then decreased as the reaction time was extended up to 30 min, suggesting there was competition for Apt between Apt-AFB_1_ adduct and Apt-Seq duplex. Interestingly, in contrast to our previous assumption, at the beginning of the 5 min reaction, Apt-Seq duplex formed from competition with Apt-AFB_1_ complex based on ascending signal. Then the Apt separated from the dsDNA duplex and formed Apt-AFB_1_ complex, gradually leading to a decrease in fluorescence after 5 min. Moreover, signals after a 5 min reaction were distinct for different concentrations of AFB_1_, demonstrating a linear relationship between AFB_1_ concentration and intensity change. Therefore, a 5 min reaction time was chosen for PG fluorescent dye and dsDNA.

### 2.5. Method Validation

Prior to quantitative analysis of AFB_1_ in real samples, detection in the presence of matrix interference was examined. A preliminary study was conducted where undiluted primary negative extract in Tris-EDTA (TE) buffer (10 mM Tris-HCl and 1 mM EDTA-2Na, pH 8.0) was mixed with a series of AFB_1_ standard solutions and fluorescence was measured at 520 nm. As shown in Figure S4, areca nut extract containing various levels of AFB_1_ displayed a negligible decrease in signal compared to the blank extract, but incurred high background levels. Based on this, various AFB_1_ levels in primary extract and TE buffer were screened using the proposed method. As observed in Figure S4, in comparison with TE buffer, TCMs extract had high background fluorescence, but this background did not obscure the decrease in signal as AFB_1_ levels increased. To deal with the complicated matrix, an approach combining dissolution in TE buffer and background correction was adopted.

**Figure 5:**
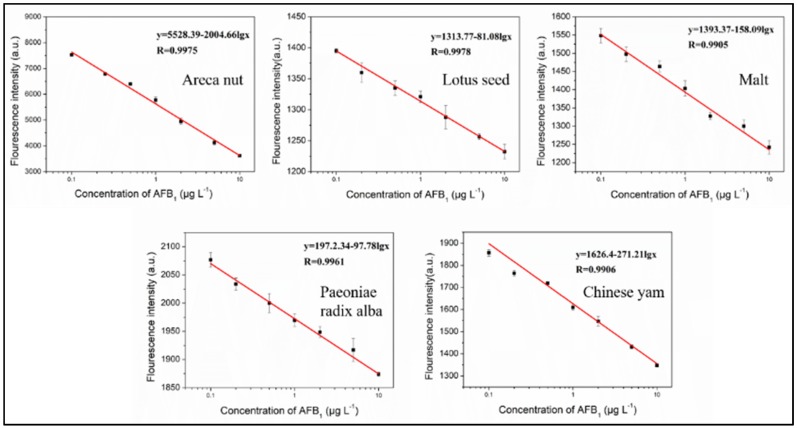
Calibration plot with relative fluorescence of PG/aptamer duplexes mixed with different concentrations of AFB_1_ in five TCMs extracts. Results are from three independent experiments.

Under the optimal conditions, various concentrations of AFB_1_ were detected using the proposed method. As shown in Figure 5, the fluorescence intensity of PG decreased gradually as AFB_1_ concentrations in the reaction system increased. A good linear relationship between the fluorescence intensity (*y*) and the logarithm of the concentration (Lg*x*) with a correlation coefficient of 0.9905–0.9976 was observed. The linear range was 0.1–10 μg·L^−1^ (0.1–10 ppb). We defined 10% fluorescence inhibition as the lowest detected concentration, thus allowing a limit of detection (LOD) of 0.1 μg·L^−1^ (coefficient of variation, CV < 2%). This suggests the AFB_1_-binding aptamer in the present study allowed for quantitative detection of AFB_1_ for levels as low as 0.1 μg·L^−1^. Recovery assays were performed where reactions were spiked with three AFB_1_ concentrations, 2.5, 5, and 10 μg·kg^−1^, which resulted in recoveries of 71.1–99.2, 78.5–100.9, and 79.4–100.4%, respectively (Table 2). Analyses were performed in triplicate. Good reproducibility was observed with reproducibility standard deviations of 2.6–10.2%, which are within acceptable levels.

### 2.6. Application to Real Samples

An edible and medicinal food, areca nut is widely distributed in Southern and Southeast Asia, including China, India, and the Philippines. Currently, it is commonly used as the main ingredient of many prescription TCMs, including to treat various gastrointestinal, parasitic, and edematous diseases, due to its wide spectrum of biological and pharmacological activities [36]. Lotus seed, which is widely used as food in China, is rich in protein, amino acids, unsaturated fatty acids, and minerals. This seed contains approximately 19.85% protein (dry weight) and has a well-balanced amino acid composition according to the Food and Agriculture Organization of the United Nations/World Health Organization with nutritive properties similar to soybean [37]. As reported in many studies, Chinese yam contains a variety of functional components, such as polysaccharides, choline, saponins, cholesterol, and phytic acid [38]. *Paeoniae alba Radix* is the *Ranunculaceae peony* root of *P. lactiflora* Pall. It has been used as an analgesic, sedative, and anti-inflammatory agent in Asia. It is also commonly used to treat patients with cardiovascular extravasated or stagnated blood [39]. However, this TCM is susceptible to toxigenic molds and the production of hazardous aflatoxin due to high temperatures and humidity where these TCMs are stored. Therefore, it is of great importance to routinely screen and measure aflatoxin contamination in TCMs prior to consumption.

The results of our study suggest our established method could be widely applicable in studies of complex medicinal herbs. To test the feasibility of our approach in real samples, five TCMs samples were collected from different regions and analyzed. As shown in Table 3, AFB_1_ was detected in six batches of the twenty tested, where three contained levels exceeding the tolerable maximum level in most foodstuffs of 5 μg·kg^−1^. To confirm this result, the suspected samples were further analyzed by liquid chromatography-electrospray tandem mass spectrometry (Appendix A). These samples were verified as positive for AFB_1_ based on two ion peaks in the extracts compared to AFB_1_ standard. The above results indicate positive samples could be determined to be AFB_1_ positive sensitively and accurately using the proposed method, demonstrating that this method is a promising rapid screening tool for AFB_1_ contamination of TCMs.

## 3. Conclusions

In summary, a simple and rapid aptamer-based label-free strategy was developed for highly selective and sensitive fluorescence detection of AFB_1_ in complicated TCMs matrices. In view of the high selectivity of aptamer to AFB_1_ and the ultra-sensitivity of PG for trace dsDNA, the proposed method accurately quantified nanomolar levels of AFB_1_. Because TCMs have complex matrices that interfere with detection of specific components, this interference was overcome by dissolution in TE buffer and background correction, largely simplifying the process. The entire detection process could be completed in 30 min. Due to its simple, label-free design, fast response, and high selectivity, the proposed method is a significant improvement in AFB_1_ screening during monitoring of TCM safety. Importantly, this is promising as a universal method due to the exceptional selectivity of PG for dsDNA and the potential extension of this principle to detecting other targets for which aptamers are available.

## 4. Materials and Methods 

### 4.1. Reagents and Chemicals

Mycotoxin standards including AFB_1_, AFM_1_, OTA, ZEN, FB_1_, and DON were purchased from Pribolab (Singapore, Singapore). A total of seven oligonucleotide sequences against AFB_1_ and each corresponding complementary ssDNA were synthesized and purified through HPLC by Sangon Biotechnology Co., Ltd. (Shanghai, China), and these aptamers were listed in Table 1 and their corresponding complementary sequences (Seq) was listed in Table S1. The fluorescent dye PicoGreen was purchased from Invitrogen (Carlsbad, CA, USA). The areca nuts, lotus seed, malt, *Radix Paeoniae Alba*, and Chinese yam were collected from China, India, Philippines, and Indonesia. Acetonitrile, methanol, Tris-HCl, EDTA-2Na, Na_2_CO_3_, NaHCO_3_, and NaCl was analytical grade and purchased from Beijing Chemical Works (Beijing, China). PestiCarb (GCB 120-400 Mesh), octadecylsilane (C18) and Cleanert primary secondary amine (PSA, 40–60 μm) were from Beijing Agela Technologies Inc. Deionized water was purified using a Milli-Q system (Millipore, CA, USA).

### 4.2. Sample Preparation

AFB_1_ was extracted from test samples using ultrasound-assisted solid-liquid extraction. Briefly, 5.0 g of each ground sample was weighed in a 50 mL centrifuge tube and 1.0 g NaCl and 25.0 mL of solvent consisting of acetonitrile/water (70/30, v/v) were added. The mixture was ultrasonicated in an ultrasonic clean bath at 500 W for 15 min and then centrifuged at 10,000 rpm for 5 min. For areca nuts, 2 mL of the supernatant was added to 0.25 g primary secondary amine(PSA), and 0.3 g graphitized carbon black(GCB). For other samples, 2 mL of supernatant was mixed with 0.25 g octadecylsilane (C18), vortexed for 2 min, and then centrifuged at 10,000 rpm for 5 min. For the areca nut samples, 1 mL supernatant was mixed with 150 μL of carbonate buffer solution (pH 9.6). This mixture was immediately transferred to a 2 mL tube and dried through evaporation under a stream of nitrogen gas at 40 °C. The residue was redissolved in TE buffer containing 10% methanol and centrifuged at 10,000 rpm for 5 min. The resulting supernatant was then used for fluorescent detection as described below.

### 4.3. Fluorescent Detection

For AFB_1_ detection, the control and test groups were tested in parallel. In the test group, 25 μL of 20 mM AFB_1_ specific-aptamer solution was mixed with 50 μL AFB_1_ standard or sample solution dissolved in TE buffer in microplate wells. These mixtures were incubated at room temperature for 20 min. Next, 25 μL of 20 mM Seq and 10 μL of 10X PG were added to each well. After incubating for 5 min, the fluorescence intensities of the wells were recorded using a multifunction microplate reader (Tecan Infinite M1000, Tecan Austria GmbH, Grödig, Austria) with an excitation of 480 nm and emission of 520 nm. The control group was tested using the same procedure as for the test group except TE buffer or negative sample solution was used in place of standard solution. Each experimental condition was tested in triplicate. M1000

## Figures and Tables

**Table 1:** DNA aptamer used in this study.

NO.	Sequence (5′–3′)	References
Apt 1	AGCAGCACAGAGGTCAGATGGTGCTATCATGCGCTCAATGGGAGACTTTAGCTGCCCCCACCTATGCGTGCTACCGTGAA	[30]
Apt 2	AGCAGCACAGAGGTCAGATGTCTAAATGACACCTTTTCAACCTACTGACTTGGTTTACTACCTATGCGTGCTACCGTGAA	[30]
Apt 3	GTTGGGCACGTGTTGTCTCTCTGTGTCTCGTGCCCTTCGCTAGGCCCACA	[31]
Apt 4	AAAGTTGGGCACGTGTTGTCTCTCTGTGTCTCGTGCCCTTCGCTAGGCCCACAAAAA	[32]
Apt 5	CAGCTTATTCAATTGTGCAGGGGGAGGGGGAGTGGTGGCTCGCGGTGCGTGGTGGCTGTAGATAGTAAGTGCAATCTA	[33]
Apt 6	GCATCACTACAGTCATTACGCATCGGGTAATCCTAAGCGGAACTGAGGAGTGGGAGGTAAATCGTGTGAAGTGCTGTCCC	[34]
Apt 7	TTTTTTGTTGGGCACGTGTTGTCTCTCTGTGTCTCGTGCCCTTCGCTAGGCCCAC	[35]

**Table 2:** Recoveries from five TCMs spiked with three concentrations of aflatoxin B_1_.

Recovery (%)	Spiking Level (μg·kg^−1^) (*n* = 3)		
	2.5	5	10
Areca nut	71.1 (2.6%)	78.9 (9.1%)	79.9 (10.2%)
Lotus seed	82.4 (4.6%)	81.4 (10.2%)	95.4 (8.7%)
Malt	75.5 (5.9%)	78.5 (1.3%)	100.4 (8.4%)
Paeoniae alba radix	99.2 (7.2%)	100.9 (4.6%)	79.4 (6.4%)
Chinese yam	89.4 (3.4%)	88.7 (5.6%)	95.5 (9.0%)

**Table 3:** Aflatoxin B_1_ contamination levels in samples.

Samples		Aflatoxin B_1_ Contaminated Level (μg·kg^−1^)	RSD (%)
Areca nut	China (Guangxi province)	ND	-
	China (Hainan province)	<LOQ	-
	India	1.1	4.6
	Indonesia	58.1	7.2
	Philippines	97.1	6.4
Lotus seed	China (Hunan province)	63.2	5.7
	China (Hubei province)	ND	-
	China (Beijing province)	ND	-
	China (Fujian province)	ND	-
Malt	China (Hebei province)	ND	-
	China (Hebei province)	0.8	4.9
	China (Hebei province)	ND	-
	China (Hebei province)	ND	-
Paeoniae alba radix	China (Zhejiang province)	ND	-
	China (Anhui province)	ND	-
	China (Anhui province)	ND	-
	China (Anhui province)	ND	-
Chinese yam	China (Henan province)	ND	-
	China (Henan province)	ND	-
	China (Henan province)	ND	-
	China (Guangxi province)	ND	-

## Supplementary Materials

The following are available online at http://www.mdpi.com/2072-6651/10/3/101/s1, Figure S1: Fluorescence intensity of aptamer binds with same length but different complementary sequence by various concentrations of AFB1, Figure S2: Optimization of the addition amount of the PicoGreen were added into the aptamer and complementary strand mixture, Figure S3: Optimization of the incubation time after aptamer complementary strand and PicoGreen were added into the different concentration of AFB1 and aptamer mixture, Figure S4: Calibration plot relative Flouresence of the PG/aptamer duplex mixture against different concentrations of AFB1 in arecae nut extract (black line, y = 5528.39 − 2004.66lgx, R = 0.9975) and TE buffer (red line, y = 5527.48 − 1976.13lgx, R = 0.9987), Table S1: DNA aptamer corresponding complementary sequence used in this study.

## Appendix A

Ultra fast liquid chromatography (UFLC)-MS/MS analysis: Sample analysis was performed using an Applied Biosystems Sciex Qtrap^®^5500 MS/MS system (Foster, CA, USA) equipped with an electrospray ionization (ESI) source coupled with a UFLC system from Shimadzu (Kyoto, Japan). The Applied Biosystems Analyst software (version 1.6.2) was used to control the UFLC-MS/MS system and for data acquisition and processing. The mycotoxins were separated on a Shiseio Capcell Core C18 column (100 × 2.1 mm I.D., 2.7 μm; Shiseio, Japan). The mobile phase consisted of (A) acetonitrile (0.1% formic acid) and (B) water (0.1% formic acid) at a flow rate of 0.3 mL·min^−1^ and the injection volume was 2 μL. The optimized gradient elution procedure was as follows: 0~0.5 min, 10~30% A; 0.5~1 min, 30% A; 1~8 min, 30~95% A; 8~10 min, 95% A; 10.01~12 min, 10% A. The mass spectrometer was operated in the positive ESI mode with MRM at unit resolution. The main MS parameters were optimized and finally set as follows: nebulizer gas (GS1), 50 psi; auxiliary gas (GS2), 50 psi; curtain gas (CUR), 35 psi; capillary temperature, 550 °C; ion spray voltage (IS), +5500 V. Nitrogen was used as the nebulizer, heater, curtain, and collision gas. The precursor-to-product ion transitions were *m*/*z* 313.1/285.1 and *m*/*z* 313.1/245.0 for AFB_1_, *m*/*z* 315.1/287.0 and *m*/*z* 315.1/259.0 for AFB_2_, *m*/*z* 329.1/243.0 and *m*/*z* 329.1/311.2 for AFG_1_, *m*/*z* 331.1/285.1 and *m*/*z* 331.1 / 241.0 for AFG_2_.
