# Supplementary material for: A Rapid Label-Free Fluorescent Aptasensor PicoGreen-Based Strategy for Aflatoxin B1 Detection in Traditional Chinese Medicines
Source: Toxins (Basel). 2018 Feb 28;10(3):101. doi: 10.3390/toxins10030101 (PMC5869389; doi:10.3390/toxins10030101)
Supplement: Supplementary file 1 [file toxins-10-00101-s001.pdf]

Supplementary Materials: A Rapid Label-Free Fluorescent Aptasensor PicoGreen-Based Strategy for Aflatoxin B1 Detection in Traditional Chinese Medicines

Cheng Zhang, Xiaowen Dou, Lei Zhang, Meifeng Sun, Ming Zhao, Zhen OuYang, Dandan Kong, F. Logrieco Antonio, and Meihua Yang

Table S1. DNA aptamer corresponding complementary sequence used in this study.

NO.	Sequence(5′–3′)	Correspond
Seq 1	TTCACGGTAGCACGCATAGGTGGGGGCAGCTAAAGTCTCCCATTG AGCGCATGATAGCACCATCTGACCTCTGTGCTGCT	Apt1
Seq 2	TTCACGGTAGCACGCATAGGTAGTAAACCAAGTCAGTAGGTTGAA AAGGTGTCAGTTAGACATCTGACCTCTGTGATGCT	Apt2
Seq 3	TGTGGGCCTAGCGAAGGGCACGAGACACAGAGAGACAACACGTG CCCAAC	Apt3
Seq 4	TTTTTGTGGGCCTAGCGAAGGGCACGAGACACAGAGAGACAACA CGTGCCCACATTT	Apt4
Seq 5	TAGATTGCACTTACTATCTACAGCCACCACGCACCGCGAGCCACC ACTCCCCCTCCCCCTGCACAATTGAATAAGCTG	Apt5
Seq 6	GGGACAGCACTTCACACGATTTACCTCCCACTCCTCAGTTCCGCTT AGGATTACCCGATGCGTAATGACTGTAGTGATGC	Apt6
Seq 7	GTGGGCCTAGCGAAGGGCACGAGACACAGAGAGACAACACGTGC CCAACAAAAAA	Apt7

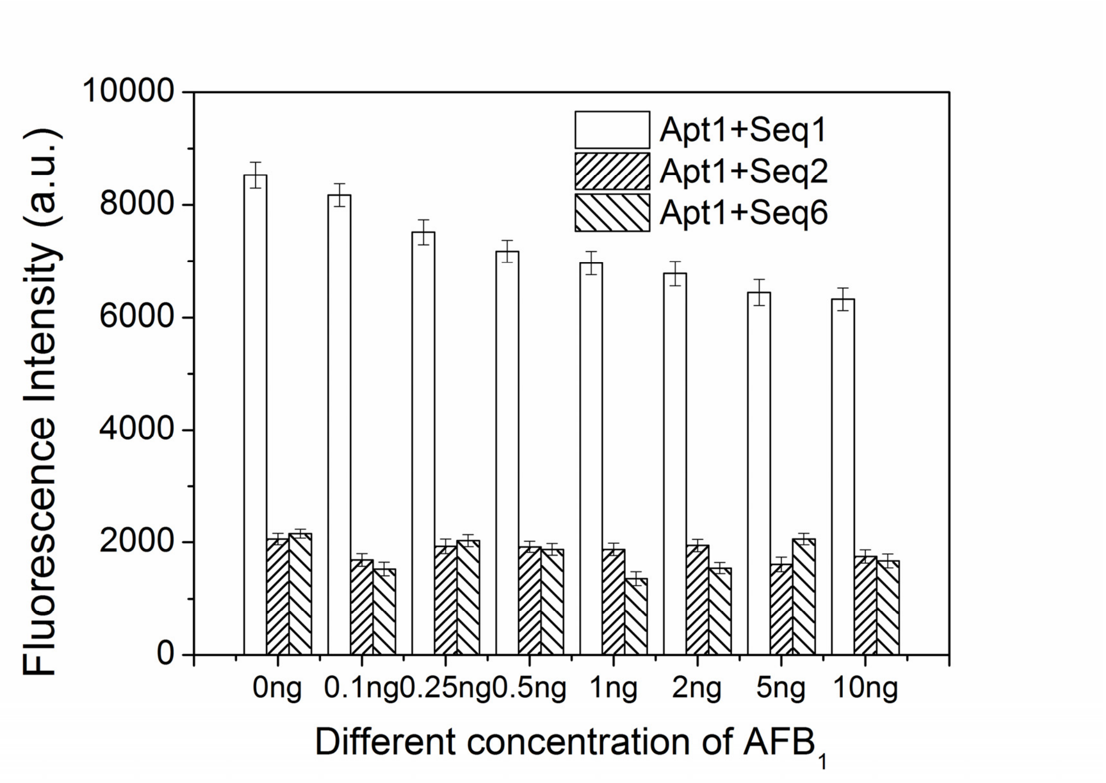

Figure S1. Fluorescence intensity of aptamer binds with same length but different complementary sequence by various concentrations of AFB1. Results are from three independent experiments.

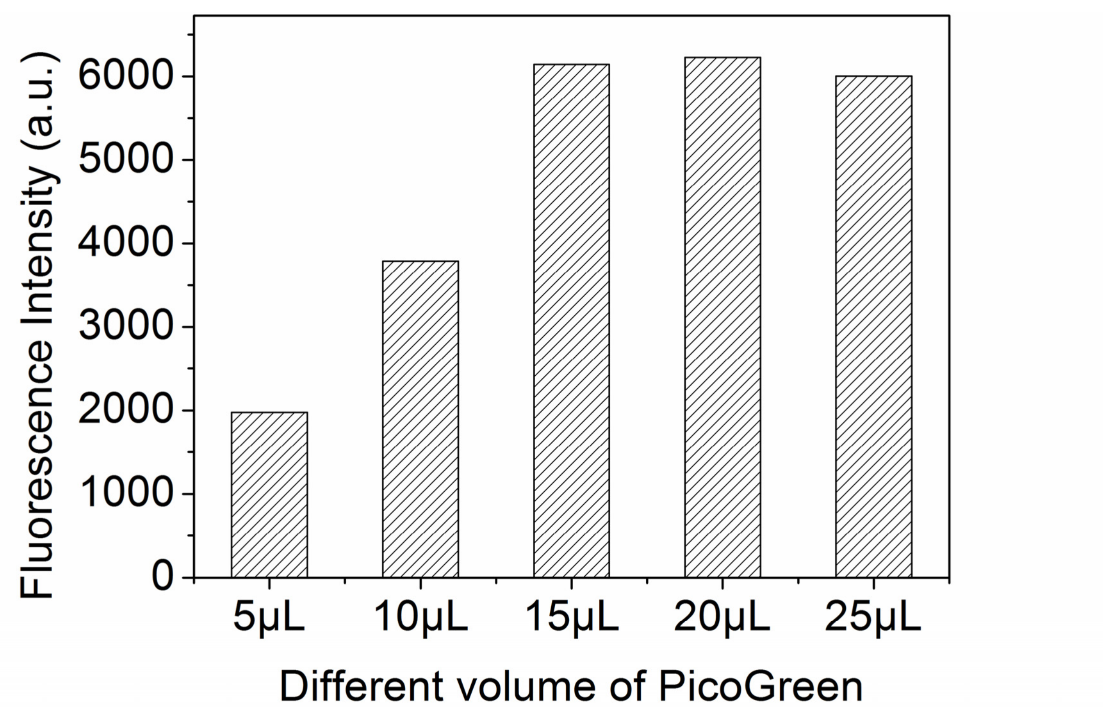

Figure S2. Optimization of the addition amount of the PicoGreen were added into the aptamer and complementary strand mixture.

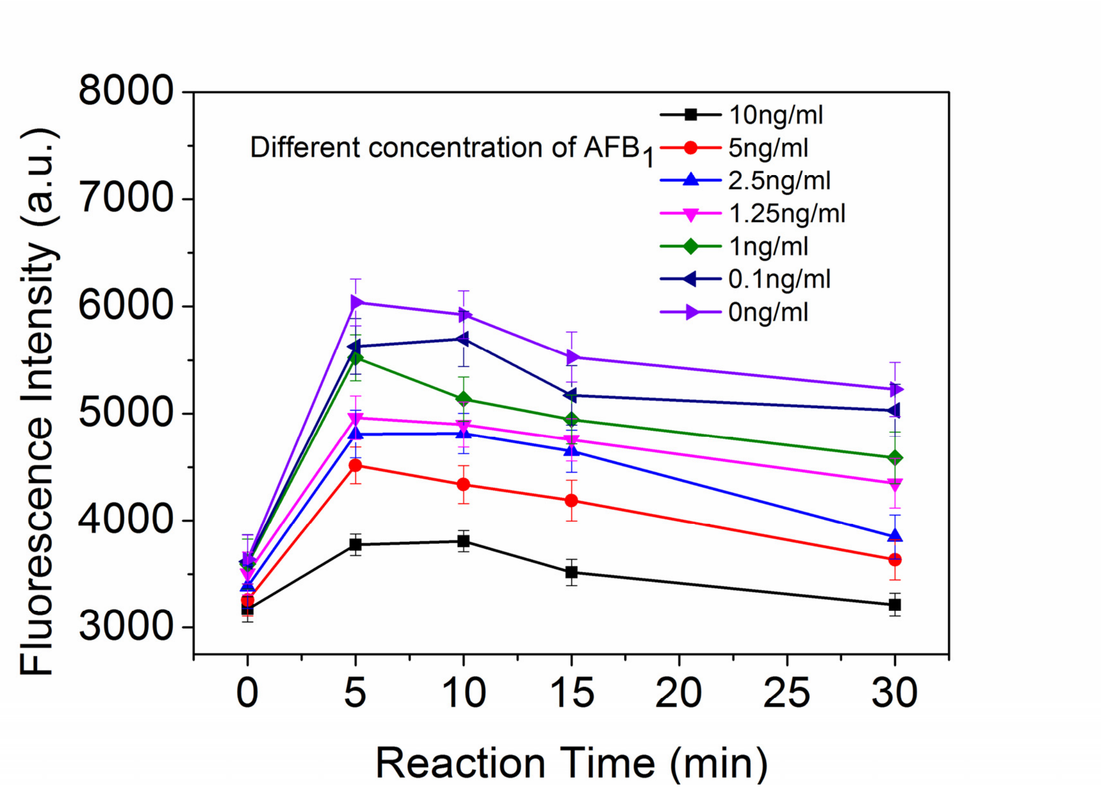

Figure S3. Optimization of the incubation time after aptamer complementary strand and PicoGreen were added into the different concentration of AFB1 and aptamer mixture. Error bars were obtained from three experiments.

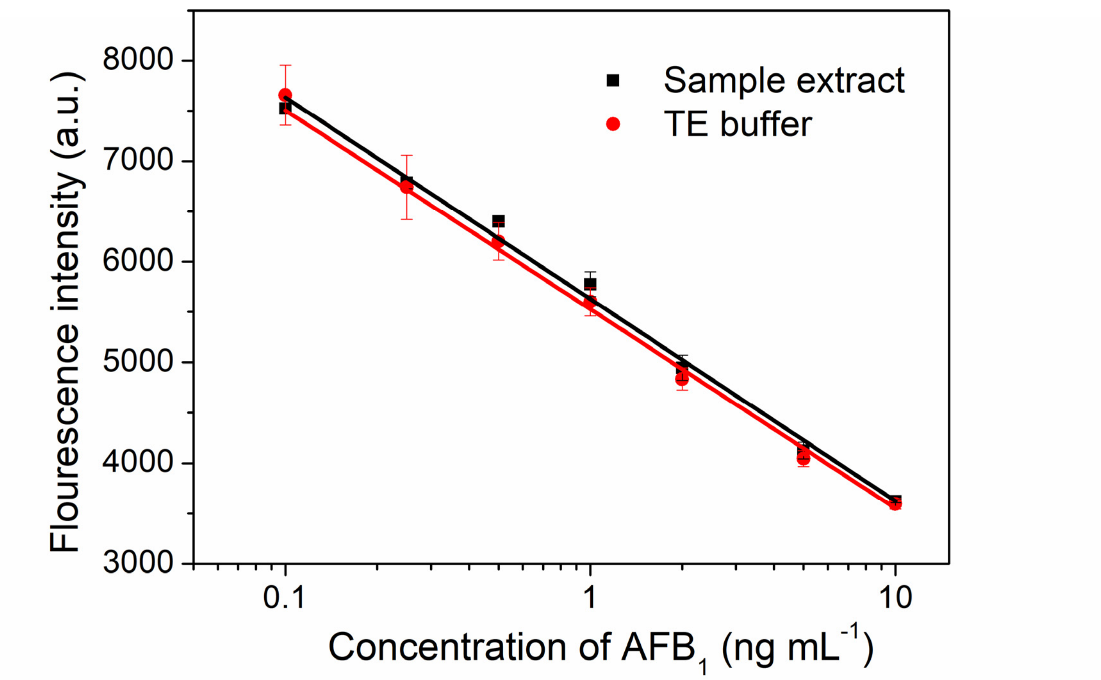

Figure S4. Calibration plot relative Flouresence of the PG/aptamer duplex mixture against different concentrations of AFB1 in arecae nut extract (black line, y=5528.39-2004.66lgx, R= 0.9975) and TE buffer (red line, y=5527.48-1976.13lgx, R=0.9987). Error bars were obtained from three experiments.
